# Miri 4-points marker: a low-cost marking device for flanged intrascleral intraocular lens fixation with modified Yamane technique

**DOI:** 10.1186/s12886-026-05134-8

**Published:** 2026-07-18

**Authors:** Yong Zheng Wai, Lee Ling Chieng, Jun Fai Yap, Nurhayati Abdul Kadir, Gim Seah Chuah, Nurul Afzan Mustapha, Lik Thai Lim, Wai Seng Chiang

**Affiliations:** 1 Department of Ophthalmology, Hospital Duchess of Kent Sandakan, Sabah, Malaysia; 2 Miri City Medical Centre, Sarawak, Malaysia; 3 Institute for Medical Research, National Institutes of Health, Selangor, Malaysia; 4 Department of Ophthalmology, Universiti Malaysia Sarawak, Kota Samarahan, Sarawak, Malaysia

**Keywords:** Astigmatism, Intraocular lens implantation, Yamane SFIOL, Scleral fixated IOL

## Abstract

**Background:**

Yamane’s flanged intrascleral intraocular lens (IOL) fixation is widely used in eyes without capsular support, but limitations in conventional conjunctival marking may affect surgical precision and consistency. This study compared the clinical outcomes of modified Yamane scleral-fixated IOL implantation using the Miri 4-Points Marker versus Castroviejo calipers, assessing visual outcomes and complications between these two approaches.

**Methods:**

This retrospective cohort study at a single hospital of Malaysia included 44 eyes operated on between 2024 and 2025. Conjunctival markings were made using either the Miri 4-Points Marker or Castroviejo calipers. Two 30-gauge thin-walled needles created right-angled sclerotomies 2 mm posterior to the limbus. IOL haptics were externalized through the needles, cauterized to form a flange, and inserted into scleral tunnels. Descriptive statistics and binary logistic regression were used to assess factors associated with postoperative visual outcomes.

**Results:**

Among 44 patients (mean age 64.4 ± 9.7 years, 77.3% male) who underwent intrascleral IOL fixation, the Miri 4-Points Marker was used in 72.7% of cases. Clinical outcomes with the Miri 4-Points Marker were comparable to Castroviejo calipers. Mean postoperative uncorrected visual acuity (UCVA) and corrected distant visual acuity (CDVA) were 0.7 (± 0.4) and 0.4 (± 0.3) logMAR, respectively. At one month postoperatively, 17.2% achieved good UCVA, increasing to 30.8% after excluding ocular comorbidities; 41.4% achieved good CDVA, rising to 46.2% without ocular comorbidities. On univariable analysis, high corneal astigmatism (> 2.5 D) was associated with poorer UCVA across all patients, regardless of Miri 4-Points Marker use (OR = 0.07, 95% CI: 0.01, 0.78, *p* = 0.03). Multivariable analysis did not identify any independent predictors.

**Conclusions:**

The Miri 4-Points Marker is a feasible and safe adjunct for conjunctival marking in Yamane intrascleral IOL fixation. Observed short-term outcomes were similar to caliper-based marking, although this study’s chronological design precludes any conclusion of equivalence.

## Background

Scleral-fixated intraocular lens (SFIOL) implantation is a feasible option for eyes without capsular bag support, with several surgical techniques described such as sutured or sutureless approaches that differ in ophthalmic outcomes [1]. In general, the transscleral sutured posterior chamber intraocular lens (IOL) offers good visual recovery but its suture-related complications namely erosion and breakage, remain significant drawbacks. For instance, breakage of 10 − 0 polypropylene sutures has been reported in 27.9% of eyes at six years postoperatively [2].

The Carlevale lens, which uses T-shaped harpoons to anchor the IOL to the sclera without sutures, represents another promising alternative [3]. Like other SFIOL techniques, it requires accurate scleral entry site localization to ensure optimal IOL centration and stable haptic positioning. Inaccurate conjunctival marking may result in sclerotomy misalignment, leading to IOL tilt, decentration or refractive error [4].

Yamane’s technique for flanged intrascleral IOL fixation is another sutureless approach that has rapidly gained popularity due to its minimally invasive nature, shorter operative time, and stable IOL positioning [5]. It can be performed using a widely available standard three-piece IOL, and its success is highly dependent on precise conjunctival marking [6]. Traditionally, conjunctival marking is performed using Castroviejo calipers in a sequential manner, which may introduce variability in inter-point distances and angular alignment. This can potentially affect the symmetry of haptic externalisation. While the Yamane double-needle stabiliser has been introduced to improve standardisation of sclerotomy site placement and needle entry alignment, it is not universally available, especially in resource-limited settings. Furthermore, ophthalmologists who do not have access to the stabiliser must continue to rely on conventional freehand caliper-based marking techniques. Therefore, there is a need for cost-effective methods that improve marking precision and reproducibility of conjunctival marking. To address this limitation, a novel surgical marking device called the Miri 4-Points Marker (manufactured by Solaris Scientific (M) Sdn Bhd, a Malaysian company) was developed. This instrument enables simultaneous creation of four equidistant conjunctival reference points at fixed 90° intervals, potentially improving consistency and reducing manual marking errors compared to conventional caliper-based techniques.

However, global evidence comparing refinements in conjunctival marking methods for SFIOL implantation remains limited despite the widespread adoption of Yamane’s technique. Most published studies have focused on overall surgical outcomes rather than specific instrument-based modifications of the marking process. For example, a recent prospective intervention study confirmed that the double‑needle flanged technique provides good IOL centration and long-term visual improvement up to three years postoperatively, but it did not specifically assess variations in marking strategies or instruments [7]. Similarly, a narrative review highlighted the lack of direct comparisons between different surgical modifications of the Yamane method, despite numerous technical refinements being described [8]. In addition, there is a lack of regional data from Malaysia, where sociodemographic profiles of patients may differ from previously reported cohorts [9]. To address these gaps, this study aimed to compare the clinical outcomes of modified Yamane SFIOL implantation using the Miri 4-Points Marker versus conventional Castroviejo calipers. The primary endpoint was the comparison of visual and refractive outcomes between the two groups. The secondary endpoints included intraoperative and postoperative complications, as well as IOL centration.

## Materials and methods

### Study design

This retrospective cohort study included all aphakic patients who underwent modified Yamane’s flanged intrascleral IOL fixation at Hospital Duchess of Kent, Sandakan in Malaysia from 1st January 2024 to 31st December 2025. Written informed consent was obtained from all patients prior to participation. The study protocol was reviewed and approved by the Medical Research & Ethics Committee of Ministry of Health Malaysia (NMRR ID 25-03848-7OA).

Patient demographics, intraoperative notes, and clinical data were retrieved from physical case records and transcribed into a standardized data collection form. The pre-operative variables of interest included age, gender, systemic comorbidities, laterality of eye, history of ocular trauma, ocular comorbidity, intraocular pressure (IOP), indication for modified Yamane’s SFIOL and prior ocular surgery. Patients younger than 18 years of age and those lost to follow-up within one month of Yamane SFIOL implantation were excluded.

The modified Yamane technique was indicated in several scenarios, including complicated cataract surgery, aphakia without capsular support, lens dislocation, and weak capsular support. Complicated cataract surgery was defined as intraoperative complications during phacoemulsification requiring conversion to intracapsular cataract extraction (ICCE) or extracapsular cataract extraction (ECCE). Aphakia without capsular support referred to cases in which elective ICCE was performed and the eye was intentionally left aphakic prior to SFIOL implantation. Lens dislocation included subluxation or dislocation of either the crystalline lens or a previously implanted IOL. Weak capsular support referred to cases in which phacoemulsification was completed but residual capsular support was inadequate for IOL implantation in the capsular bag or sulcus.

In addition, intraoperative parameters such as corneal wound size, duration of surgery, type of marking tool used, and additional surgical procedures were documented. Postoperative outcomes including IOP, one-month uncorrected visual acuity (UCVA), one-month corrected distant visual acuity (CDVA), corneal astigmatism, and complications were also recorded.

### Surgical procedure

All surgeries were performed by four ophthalmologists at the hospital. Patients were listed for secondary SFIOL implantation using the modified Yamane’s technique, and none of the cases involved combined cataract extraction and SFIOL implantation in the same setting. All procedures were performed under peribulbar anesthesia. The conjunctival marking process differed depending on the instrument used. Although both horizontal and vertical quadrant fixation sites have been described in the literature for SFIOL implantation [10], available comparative retrospective study indicates that flanged intrascleral haptic fixation in both planes yields similar safety in terms of visual acuity, refractive outcomes, anterior segment parameters, and IOL centralization, with no significant differences in overall complication rates apart from a slightly higher incidence of conjunctival involvement in the vertical group. Vertical quadrant fixation may be more ergonomic for surgeons operating temporally, whereas surgeons positioned superiorly may find the horizontal approach more convenient during SFIOL implantation. As all four surgeons in our centre routinely operate from the superior position and in keeping with institutional practice as well as available evidence, the horizontal quadrant approach was consistently adopted throughout this study. Before June 2024, cases were marked using Castroviejo calipers, after which the Miri 4-Points Marker replaced it for all cases undergoing secondary SFIOL implantation. For cases using Castroviejo calipers, three cardinal lines were first marked at 12, 3, and 9 o’clock using a toric reference marker. The first mark was then placed 2 mm posterior to the limbus on the 3 o’clock reference line, followed by a second mark positioned 2 mm superior to the first in a perpendicular fashion. Similarly, a third mark was placed 2 mm posterior to the limbus on the 9 o’clock reference line, and a fourth mark 2 mm inferior to the third in the same perpendicular manner [6]. A distance of 2 mm from the limbus was used, in accordance with the original flanged intrascleral fixation technique described by Shin Yamane [6].

**Fig. 1:**
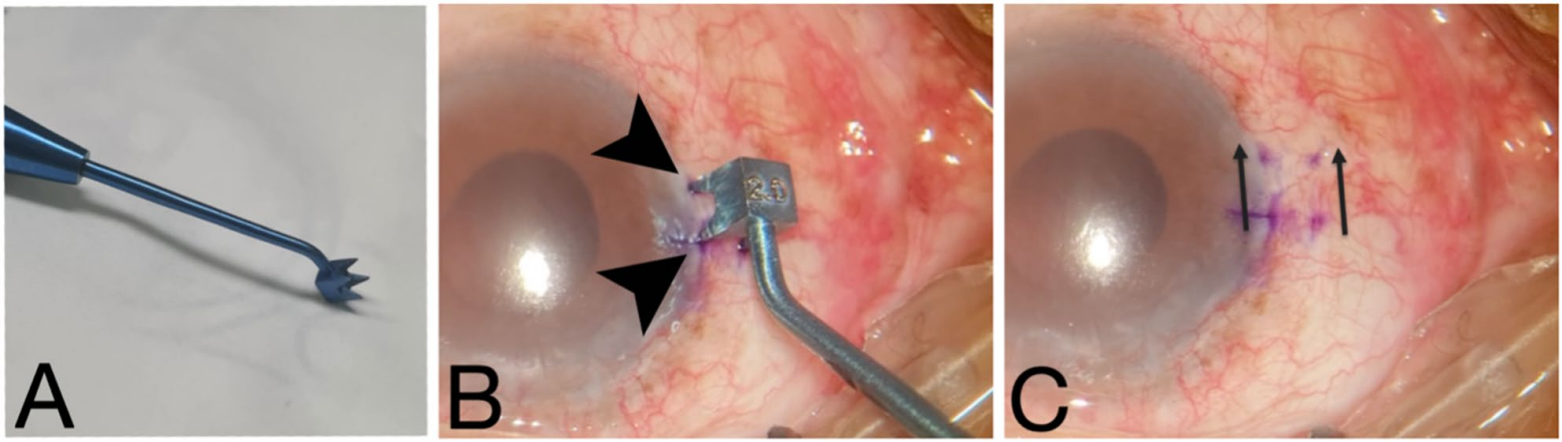
(**A**) Close-up of the Miri 4-Points Marker demonstrating four sharp tips spaced 2 mm apart. (**B**) Application of the marker at the limbus (arrowheads), with one point aligned to the 9 o’clock cardinal meridian. (**C**) Resulting four limbal marks, with the temporal pair positioned parallel to the limbus (arrows)

For patients in whom the Miri 4-Points Marker was used, four equidistant marks were created simultaneously. The device produced two conjunctival marks and two limbal reference points at the 3 o’clock position, thereby creating two sclerotomy sites perpendicular to each other. The same process was then repeated on the 9 o’clock reference line, resulting in four precise 90-degree sclerotomy marks (Fig. 1). Following the conjunctival markings, side ports were created, and an anterior chamber maintainer was inserted. A 2.75 mm blade was used to fashion the main incision.

Subsequently, an angled sclerotomy was created near the 3 o’clock position according to the predefined marks using a 30-gauge thin-wall needle (TSK ultra-thin wall needle; Tochigi Seiko, Tochigi, Japan). A three-piece IOL PU6AC (Kowa, Tokyo, Japan) was introduced into the anterior chamber through the main incision. The leading haptic was engaged with the needle tip and partially threaded into the needle lumen before the IOL was fully injected, with the trailing haptic deliberately kept outside to prevent the IOL from dislocating into the vitreous. The remainder of the leading haptic was advanced into the needle lumen with microforceps until approximately 80% of the haptic was secured, after which the needle was withdrawn and the haptic externalized onto the conjunctiva. The externalized haptic was cauterized with low-temperature cautery to create a flange. A second sclerotomy was then made at the 9 o’clock position, and the trailing haptic was inserted, externalized, and flanged in the same manner. Both flanges were subsequently tucked into the scleral tunnels to secure the IOL.

### Variable measurement

All patients were reviewed on postoperative day 1, day 7, and at one month after surgery. Corneal keratometry, UCVA, and CDVA were measured by an optometrist at the one-month postoperative visit. The presence of lens tilt was assessed using slit-lamp examination, as anterior segment optical coherence tomography was not available at our centre. Lens tilt was identified by observing asymmetry in the IOL position relative to the visual axis, such as uneven vaulting of the optic or decentration of the IOL edges. Dynamic examination with varying illumination angles and patient gaze positions was also performed to enhance visualization of subtle tilt. Cases with clinically appreciable misalignment of the IOL optic plane were recorded as having IOL tilt. In cases where lens tilt was detected clinically, research has shown the mean measured angle to be approximately 12.8 ± 3.9 degree [11]. However, this value was derived from a different patient population and is not based on our study cohort. IOP was measured using Goldmann applanation tonometry at all visits. Any elevation in IOP requiring anti-glaucoma medication, in patients without a prior history of glaucoma, was considered a postoperative complication. Other complications were also documented during the three follow-up visits.

### Statistical analysis

Descriptive statistics were used to summarize baseline demographic and clinical characteristics. Continuous variables were presented as mean (± standard deviation), while categorical variables were expressed as frequencies and percentages.

Comparisons of outcomes between groups were performed using univariable analysis. Odds ratios (OR) with 95% confidence intervals (CI) were calculated to assess associations between potential predictors and postoperative visual outcomes, including UCVA and CDVA. To address zero cell counts for selected variables of interest in contingency tables, a continuity correction using the Haldane-Anscombe method was applied by adding 0.5 to all cells prior to calculation of OR.

Univariable analyses were first performed to assess potential factors associated with postoperative visual outcomes, and variables with *p* < 0.25 were entered into the final multivariable logistic regression model to identify independent predictors. Model performance was evaluated using overall classification accuracy and Nagelkerke R². Subgroup analysis was performed among patients without ocular comorbidities or preoperative glaucoma. A p-value of < 0.05 was considered statistically significant in the final model.

## Results

### Baseline characteristics

A total of 44 patients (mean age 64.4 ± 9.7 years; 77.3% male) underwent intrascleral IOL fixation at our centre between January 2024 and December 2025. Baseline characteristics are summarized in Tables 1 and 2.

Ocular comorbidities were present in 22 eyes (50.0%). Lens-induced glaucomas were among the most commonly observed conditions, particularly phacolytic glaucoma (9.7%) and phacomorphic glaucoma (6.5%). Other preoperative glaucoma-related conditions included pseudoexfoliation glaucoma, angle-recession glaucoma and pigment dispersion syndrome. Non-glaucomatous ocular comorbidities were also identified, including corneal pathologies such as corneal scarring and decompensated cornea, as well as retinal abnormalities namely diabetic macular edema, cystoid macular edema, extrafoveal scar, and idiopathic polypoidal choroidal vasculopathy.

The most common indications for the Yamane technique were complicated cataract surgery (36.4%) and aphakia without capsular support (31.8%). Additional surgical procedures were performed in only two eyes (4.5%), including iridodialysis repair with pupilloplasty in one eye and pupilloplasty alone in another. The remaining 42 eyes underwent SFIOL implantation without concomitant surgery.

The mean operative time was 38.2 (± 24.0) minutes, and 35.4 (± 20.1) minutes when excluding the two cases that required additional intraoperative procedures. The mean postoperative UCVA was 0.7 (± 0.3) logMAR, whereas the mean postoperative CDVA further improved to 0.4 (± 0.3) logMAR.

A total of four patients (10.3%) developed postoperative complications. Two patients (5.1%) had ocular hypertension requiring anti-glaucoma medications. One patient (2.6%) had iris captured by the IOL, and one patient (2.6%) developed cystoid macular oedema.

**Table 1:** Baseline characteristics of patients who underwent scleral-fixated intraocular lens implantation between January 2024 and December 2025 (*n* = 44)

	Count (*n*)	Percentage (%)
**Gender (*n*** **= 44)**		
Female	10	22.7
Male	34	77.3
**Presence of medical illness (** ***n*** ** = 44)**		
No	8	18.2
Yes	36	81.8
**- Diabetes (** ***n*** ** = 44)**		
No	30	68.2
Yes	14	31.8
**- Hypertension (** ***n*** ** = 44)**		
No	12	27.3
Yes	32	72.7
**- Dyslipidemia (** ***n*** ** = 44)**		
No	29	65.9
Yes	15	34.1
**Laterality of eye (** ***n*** ** = 44)**		
Left eye	24	54.5
Right eye	20	45.5
**History of trauma on that eye (** ***n*** *** = 44)***		
No	33	75.0
Yes	11	25.0
**Indications for the Yamane technique (** ***n*** *** = 44)***		
Aphakia without capsular support	14	31.8
Complicated cataract surgery	16	36.4
Lens dislocation	8	18.2
Weak capsular support	6	13.6
**History of trans pars plana vitrectomy (** ***n*** ** = 44)**		
No	34	77.3
Yes	10	22.7
**Size of corneal wound (** ***n*** ** = 44)**		
Big (≥ 3 mm)	29	65.9
Small (< 3 mm)	15	34.1
**Ocular comorbidity (** ***n*** ** = 44)**		
No	22	50.0
Yes	22	50.0
**Use of Miri 4-Points Marker (** ***n*** ** = 44)**		
No	12	27.3
Yes	32	72.7
**Clinically significant corneal astigmatism (** ***n*** ** = 34)**		
No (≤ 0.75 D)	2	5.9
Yes (> 0.75 D)	32	94.1
**High corneal astigmatism (** ***n*** ** = 34)**		
No (≤ 2.5 D)	8	23.5
Yes (> 2.5 D)	26	76.5
**Complications postoperatively (** ***n*** ** = 39)**		
No	35	89.7
Yes	4	10.3
**Lens tilt (** ***n*** ** = 41)**		
No	35	85.4
Yes	6	14.6
**Postoperative uncorrected visual acuity (** ***n*** ** = 40)**		
Poor (> 6/12)	34	85.0
Good (≤ 6/12)	6	15.0
**Postoperative best-corrected visual acuity (** ***n*** ** = 40)**		
Poor (> 6/12)	21	52.5
Good (≤ 6/12)	19	47.5

**Table 2:** Baseline characteristics of patients who underwent scleral-fixated intraocular lens implantation between January 2024 and December 2025 (*n* = 44)

	Mean (± Standard deviation)
Age (years) (*n* = 44)	64.4 (± 9.7)
Duration of operation (minutes) (*n* = 43)	38.2 (± 24.0)
Intraocular pressure pre-operatively (mmHg) (*n* = 36)	17.6 (± 7.9)
Intraocular pressure one month post-operatively (mmHg) (*n* = 42)	16.0 (± 4.0)
Postoperative uncorrected visual acuity (logMAR) (*n* = 40)	0.7 (± 0.3)
Postoperative best-corrected visual acuity (logMAR) (*n* = 39)	0.4 (± 0.3)
Postoperative best-corrected visual acuity without ocular comorbidity (logMAR) (*n* = 18)	0.24 (± 0.27)
K1 (D) (*n* = 34)	41.5 (± 2.7)
K2 (D) (*n* = 34)	46.7 (± 2.1)
Corneal astigmatism (D) (*n* = 34)	5.3 (± 3.3)

### Miri 4-points marker

In eyes undergoing intrascleral fixation using the Miri 4-Points Marker (*N* = 32), the mean postoperative UCVA and CDVA were 0.7 (± 0.4) and 0.4 (± 0.3) logMAR, respectively. At one month postoperatively, 17.2% of eyes achieved good UCVA, increasing to 30.8% after exclusion of ocular comorbidities. Similarly, 41.4% attained good CDVA, which improved to 46.2% in eyes without comorbidities when Miri 4-Points Marker was used. The surgical outcomes using the Miri 4-Points Marker were comparable to those of the conventional marking method, with no statistically significant differences observed across intraoperative characteristics, postoperative visual outcomes, or complication rates. For instance, mean operation time was 38.9 (± 14.2) minutes with the Castroviejo calipers versus 34.1 (± 21.9) minutes with the Miri 4-Points Marker, but this difference was not statistically significant.

At univariable level, none of the evaluated factors were significantly associated with postoperative UCVA. Descriptively, all (100%) eyes with high corneal astigmatism (> 2.5 D) and larger corneal wounds had poor UCVA outcomes. Lens tilt and postoperative complications were also exclusively observed in the poor UCVA group.

In a subgroup analysis of patients who underwent intrascleral lens fixation using the Miri 4-Points Marker (*N* = 32), none of the variables were independently associated with postoperative CDVA in the multivariable logistic regression model adjusted for hypertension, laterality, corneal wound size, and high corneal astigmatism (Data not shown).

### Uncorrected visual acuity (UCVA)

Approximately 15.0% of patients achieved good UCVA (6/12 or better) at 1 month following scleral-fixated IOL implantation using the modified Yamane technique. This proportion increased to 27.8% after excluding patients with ocular comorbidities. On univariable level, high corneal astigmatism (> 2.5 D) was significantly associated with poorer postoperative UCVA (Table 3). Patients with high corneal astigmatism had significantly lower odds of achieving good UCVA compared with those with ≤ 2.5 D astigmatism (OR = 0.07, 95% CI: 0.01, 0.78, *p* = 0.03).

Other variables, including age, duration of operation, lens dislocation, and history of trans pars plana vitrectomy, demonstrated borderline associations but did not reach statistical significance. Multivariable logistic regression analysis was not performed due to the presence of zero cell counts in several variables, which would result in unstable model estimates. After applying a 0.5 continuity correction to account for zero counts in the large corneal wound group, the odds of achieving good postoperative UCVA were approximately 48 times higher in patients with small corneal wounds compared to those with large wounds (OR ≈ 47.7) (Haldane-Anscombe method).

**Table 3:** Factors associated with postoperative uncorrected visual acuity among patients who underwent scleral-fixated intraocular lens implantation (*n* = 44)

	Postoperative uncorrected visual acuity (UCVA)		Crude odds ratio (95% CI)	*p*-value
	Poor (Worse than 6/12)	Good (6/12 or better)		
	Count (Row %)	Count (Row %)		
**Gender (** ***n*** ** = 40)**				
Female	9 (100)	0 (0)	*1.00 (Reference)*	
Male	25 (80.6)	6 (19.4)	-	-
**Diabetes (** ***n*** ** = 40)**				
No	22 (81.5)	5 (18.5)	*1.00 (Reference)*	
Yes	12 (92.3)	1 (7.7)	0.37 (0.04, 3.51)	0.38
**Hypertension (** *n* ** = 40)**				
No	11 (100)	0 (0)	*1.00 (Reference)*	
Yes	23 (79.3)	6 (20.7)	**-**	**-**
**Dyslipidemia (** *n* ** = 40)**				
No	22 (88.0)	3 (12.0)	*1.00 (Reference)*	
Yes	12 (80.0)	3 (20.0)	1.83 (0.32, 10.53)	0.50
**Laterality of eye (** *n* ** = 40)**				
Left eye	19 (90.5)	2 (9.5)	*1.00 (Reference)*	
Right eye	15 (78.9)	4 (21.1)	2.53 (0.41, 15.75)	0.32
**History of trauma on that eye (** *n* ** = 40)**				
No	26 (86.7)	4 (13.3)	*1.00 (Reference)*	
Yes	8 (80.0)	2 (20.0)	1.63 (0.25, 10.58)	0.61
**Indications for the Yamane technique (** *n* ** = 40)**				
Aphakia without capsular support	13 (92.9)	1 (7.1)	*1.00 (Reference)*	
Complicated cataract surgery	13 (92.9)	1 (7.1)	1.00 (0.06, 17.75)	1.00
Lens dislocation	4 (57.1)	3 (42.9)	9.75 (0.78, 121.84)	0.08
Weak capsular support	4 (80.0)	1 (20.0)	3.25 (0.16, 64.61)	0.44
**History of trans pars plana vitrectomy (** *n* ** = 40)**				
No	29 (93.5)	2 (6.5)	*1.00 (Reference)*	
Yes	5 (55.6)	4 (44.4)	11.60 (1.66, 81.10)	0.20
**Size of corneal wound (** *n* ** = 40)**				
Big (≥ 3 mm)	27 (100.0)	0 (0)	*1.00 (Reference)*	
Small (< 3 mm)	7 (53.8)	6 (46.2)	-	**-**
**Ocular comorbidity (** ***n*** ** = 40)**				
No	7 (38.9)	11 (61.1)	*1.00 (Reference)*	
Yes	14 (63.6)	8 (36.4)	0.36 (0.10, 1.32)	0.12
**Use of Miri 4-Points Marker** **(** *n* **= 40)**				
No	10 (90.9)	1 (9.1)	*1.00 (Reference)*	
Yes	24 (82.8)	5 (17.2)	2.08 (0.22, 20.17)	0.53
**Clinically significant corneal astigmatism (** *n* ** = 34)**				
No (≤ 0.75 D)	1 (50.0)	1 (50.0)	*1.00 (Reference)*	
Yes (> 0.75 D)	29 (90.6)	3 (9.4)	0.10 (0.01, 2.11)	0.14
**High corneal astigmatism (** ***n*** ** = 34)**				
No (≤ 2.5 D)	5 (62.5)	3 (37.5)	*1.00 (Reference)*	
Yes (> 2.5 D)	25 (96.2)	1 (3.8)	0.07 (0.01, 0.78)	**0.03**
**Complications postoperatively (** ***n*** ** = 38)**				
No	30 (85.7)	5 (14.3)	*1.00 (Reference)*	
Yes	2 (66.7)	1 (33.3)	3.00 (0.23, 39.61)	0.40
**Lens tilt (** ***n*** ** = 40)**				
No	28 (82.4)	6 (17.6)	*1.00 (Reference)*	
Yes	6 (100)	0 (0)	-	-
Age (*n* = 40)	-	-	0.92 (0.83, 1.01)	0.08
Duration of operation (*n* = 39)	-	-	0.89 (0.79, 1.01)	0.07
**Intraocular pressure pre-operatively** **(** ***n*** **= 32)**	-	-	0.97 (0.85, 1.10)	0.62
**Intraocular pressure one month post-operatively** **(** ***n*** **= 42)**	-	-	1.05 (0.85, 1.29)	0.66

### Corrected distant visual acuity (CDVA)

A total of 47.5% of patients achieved good CDVA (6/12 or better) at 1 month post procedure. The percentage further increased to 61.1% after excluding patient with ocular comorbidities.

In the multivariable logistic regression model (adjusted for hypertension, laterality of eye, history of trans pars plana vitrectomy, size of corneal wound, ocular comorbidity, use of Miri 4-Points Marker, high corneal astigmatism, and age), none of the included variables were independently associated with postoperative CDVA (Table 4). The final model demonstrated an overall classification accuracy of 52.9%, with a Nagelkerke R² of 0.438, indicating that the included variables collectively explained 43.8% of the variation in postoperative CDVA.

**Table 4:** Factors associated with postoperative corrected distant visual acuity among patients who underwent scleral-fixated intraocular lens implantation (*N* = 44)

	Postoperative corrected distant visual acuity (CDVA)		Crude odds ratio (95% CI)	*p*-value	Adjusted odds ratio (95% CI)	*p*-value
	Poor (> 6/12)	Good (≤ 6/12)				
	Count (Row %)	Count (Row %)				
**Gender (** ***n*** ** = 40)**						
Female	6 (66.7)	3 (33.3)	*1.00 (Reference)*			
Male	15 (48.4)	16 (51.6)	2.13 (0.45, 10.10)	0.34	-	-
**Diabetes (** ***n*** ** = 40)**						
No	14 (51.9)	13 (48.1)	*1.00 (Reference)*			
Yes	7 (53.8)	6 (46.2)	0.92 (0.25, 3.48)	0.91	-	-
**Hypertension (** ***n*** ** = 40)**						
No	8 (72.7)	3 (27.3)	*1.00 (Reference)*			
Yes	13 (44.8)	16 (55.2)	3.28 (0.72, 14.94)	**0.12**	2.16 (0.22, 20.93)	0.51
**Dyslipidemia (** ***n*** ** = 40)**						
No	11 (44.0)	14 (56.0)	*1.00 (Reference)*			
Yes	10 (66.7)	5 (33.3)	0.39 (0.10, 1.49)	0.33	-	-
**Laterality of eye (** ***n*** ** = 40)**						
Left eye	14 (66.7)	7 (33.3)	*1.00 (Reference)*			
Right eye	7 (36.8)	12 (63.2)	3.43 (0.93, 12.59)	**0.06**	5.19 (0.71, 38.11)	0.11
**History of trauma on that eye (** ***n*** ** = 40)**						
No	17 (56.7)	13 (43.3)	*1.00 (Reference)*			
Yes	4 (40.0)	6 (60.0)	1.96 (0.46, 8.42)	0.37	-	-
*Indications for the Yamane technique ( ***n*** ** = 40)**						
Aphakia without capsular support	9 (64.3)	5 (35.7)	*1.00 (Reference)*			
Complicated cataract surgery	7 (50.0)	7 (50.0)	1.80 (0.40, 8.18)	0.45	-	-
Lens dislocation	2 (28.6)	5 (71.4)	4.50 (0.63, 32.30)	**0.14**	-	-
Weak capsular support	3 (60.0)	2 (40.0)	1.20 (0.15, 9.77)	0.87	-	-
**History of trans pars plana vitrectomy (** ***n*** ** = 40)**						
No	18 (58.1)	13 (41.9)	*1.00 (Reference)*			
Yes	3 (33.3)	6 (66.7)	2.77 (0.58, 13.16)	**0.20**	7.32 (0.43, 125.93)	0.17
**Size of corneal wound (** ***n*** ** = 40)**						
Big (≥ 3 mm)	16 (59.3)	11 (40.7)	*1.00 (Reference)*			
Small (< 3 mm)	5 (38.5)	8 (61.5)	2.33 (0.60, 9.03)	**0.22**	0.09 (0.002, 4.73)	0.09
**Ocular comorbidity (** ***n*** ** = 40)**						
No	7 (38.9)	11 (61.1)	*1.00 (Reference)*			
Yes	14 (63.6)	8 (36.4)	0.36 (0.10, 1.32)	**0.12**	0.85 (0.14, 5.22)	0.86
**Use of Miri 4-Points Marker (** ***n*** ** = 40)**						
No	4 (36.4)	7 (63.6)	*1.00 (Reference)*			
Yes	17 (58.6)	12 (41.4)	0.40 (0.10, 1.69)	**0.22**	0.60 (0.07, 4.85)	0.63
**Clinically significant corneal astigmatism (** ***n*** ** = 34)**						
No (≤ 0.75 D)	0 (0)	2 (100)	*1.00 (Reference)*			
Yes (> 0.75 D)	18 (56.3)	14 (43.8)	-	-	-	**-**
**High corneal astigmatism (** ***n*** ** = 34)**						
No (≤ 2.5 D)	2 (25.0)	6 (75.0)	*1.00 (Reference)*			
Yes (> 2.5 D)	16 (61.5)	10 (38.5)	0.21 (0.04, 1.24)	**0.09**	0.06 (0.002, 1.56)	0.09
**Complications postoperatively (** ***n*** ** = 38)**						
No	18 (51.4)	17 (48.6)	*1.00 (Reference)*			
Yes	2 (66.7)	1 (33.3)	0.53 (0.04, 6.39)	0.62	-	-
**Lens tilt (** ***n*** ** = 40)**						
No	17 (50.0)	17 (50.0)	*1.00 (Reference)*			
Yes	4 (66.7)	2 (33.3)	0.50 (0.08, 3.10)	0.46	-	-
**Age (** ***n*** ** = 40)**	-	-	0.96 (0.90, 1.03)	**0.22**	0.91 (0.80, 1.04)	0.17
**Duration of operation (** ***n*** ** = 39)**	-	-	0.99 (0.96, 1.02)	0.36	-	-
**Intraocular pressure pre-operatively (** ***n*** ** = 32)**	-	-	0.97 (0.89, 1.06)	0.54	-	-
**Intraocular pressure one month post-operatively (** ***n*** ** = 42)**	-	-	0.96 (0.82, 1.12)	0.59	-	-

The final multivariable logistic regression model was adjusted for hypertension, laterality of eye, history of trans pars plana vitrectomy, size of corneal wound, ocular comorbidity, use of Miri 4-Points Marker, high corneal astigmatism and age

* The variable ‘indications for the Yamane technique’ was not included in the multivariable logistic regression despite one subcategory demonstrating *p* < 0.25 in univariable analysis. This was due to the small number of cases within several categories, resulting in sparse data and unstable parameter estimates, as reflected by wide confidence intervals. Inclusion of this variable could lead to model overfitting given the limited sample size

### Subgroup analysis of patients without ocular comorbidity or pre-operative glaucoma

Among patients without ocular comorbidity or pre-operative glaucoma who underwent intrascleral lens fixation (*N* = 22), no factors were found to be significantly associated with postoperative CDVA, as all variables demonstrated p-values greater than 0.05 (Data not shown).

Several variables, including Miri 4-Points Marker, clinically significant corneal astigmatism, and postoperative UCVA, could not be analyzed inferentially due to zero cell counts. Nevertheless, descriptive analysis demonstrated that all patients with good postoperative UCVA achieved good CDVA. Interpretation of these findings is limited by the small sample size and wide confidence intervals.

## Discussion

Overall, satisfactory postoperative visual acuity was achieved in the group (*n* = 32) utilizing Miri 4-Points Marker, particularly in eyes without ocular comorbidities. CDVA improved from 0.7 to 0.4 LogMAR. This improvement in CDVA appears more pronounced than that reported in a previous Malaysian study on ICCE, although such differences should be interpreted with caution given the fundamental variations in surgical approach, patient selection, and perioperative context between the two cohorts. The observed disparity may also be partly attributable to differences in sample size [12]. Notably, higher corneal astigmatism was associated with poorer UCVA, highlighting the importance of postoperative corneal status in visual rehabilitation.

The comparable outcomes observed between the Miri 4-Points Marker and the conventional technique suggest that, although the Yamane method itself is inherently stable and reproducible, the marking approach may play a supportive rather than determinant role in final visual outcomes. The present findings are consistent with previous studies demonstrating good visual and anatomical outcomes with this technique [13]. However, most existing literature has focused on the core surgical method, with limited attention to refinements in marking strategies.

Accurate sclerotomy placement remains a critical step in achieving optimal IOL centration and minimizing tilt. Conventional marking with Castroviejo calipers requires sequential measurements, which may introduce inter-point variability, particularly in maintaining a precise 90° relationship between entry sites. In contrast, the Miri 4-Points Marker allows simultaneous placement of equidistant points, potentially improving geometric accuracy and reproducibility. While this theoretical advantage did not translate into statistically superior visual outcomes in our cohort, it may reduce variability and facilitate consistency, especially among ophthalmologists early in their learning curve.

A fixation distance of 2 mm posterior to the limbus was selected for several key reasons. First, this measurement adheres to the original technique described by Yamane. Optically, the 2 mm distance provides a more predictable effective lens position (ELP). Literature demonstrates that flanged intrascleral fixation at 2 mm yields an ELP approximately 0.62 mm more posterior than primary intracapsular implantation [13]. Conversely, extending the fixation distance to 2.5 mm shifts the ELP even further posteriorly, which fails to induce a compensatory myopic shift and frequently results in hyperopic refractive errors. Anatomically, 2 mm distance is associated with a significantly lower risk of postoperative cystoid macular edema compared to 2.5 mm and 3.0 mm markings [14]. Therefore, the 2 mm parameter was chosen to optimize refractive outcomes and minimize macular complications.

The influence of ocular comorbidities on postoperative outcomes observed in this study is consistent with prior reports. Eyes without coexisting pathology achieved better visual outcomes, reflecting the limiting effects of conditions such as corneal disease, glaucoma, and macular pathology on visual recovery [15]. Importantly, patients with ocular comorbidities were not excluded in order to reflect real-world clinical practice. This is particularly relevant in our population, where late presentations and complex ocular histories are common, especially in resource-limited settings.

The IOL utilized in this study features a three-piece design incorporating polyvinylidene fluoride (PVDF) haptics, which offer distinct biomechanical advantages over traditional polymethyl methacrylate (PMMA) alternatives. While PMMA has historically provided high structural rigidity and excellent dimensional stability within the intact capsular bag, its glassy polymer composition yields a high flexural modulus, rendering it susceptible to permanent plastic deformation, kinking, or snapping when subjected to excessive intraoperative manipulation [16]. Conversely, PVDF is a highly flexible, semi-crystalline fluoropolymer characterized by superior elasticity and “loop memory,” allowing the haptic arms to resiliently recover their engineered configuration after compressed delivery or surgical folding. Furthermore, in advanced sutureless scleral fixation techniques, PVDF undergoes uniform thermal melting to produce a stable, mushroom-shaped flange that achieves a significantly higher mean pull-out dislocation force (approximately 1.58 N to 2.04 N) compared to the sharp, conical flanges formed by PMMA (0.70 N), thereby reducing the risk of haptic breakage and long-term lens subluxation [17, 18]. Furthermore, in vitro comparative studies of secondary IOL fixation techniques have demonstrated that the flanged haptic (Yamane) technique provides the highest resistance to axial traction compared with other scleral fixation methods, including transscleral tunnels, glued haptics, and bent haptic techniques, indicating overall fixation strength [19]. Within this context, the higher dislocation forces observed with PVDF flanges further support their biomechanical advantage in achieving secure intrascleral fixation in Yamane-type procedures.

A key finding of this study was the significant association between high corneal astigmatism (> 2.5 D) and poorer UCVA. This is clinically expected, as uncorrected refractive error directly impacts visual acuity despite adequate IOL positioning. The high astigmatism observed may be partly attributable to the large corneal wounds (≥ 3 mm) in a substantial proportion of patients (65.9%). Similar observations have been reported in studies evaluating refractive outcomes following secondary IOL implantation [20]. Notably, CDVA was not significantly associated with corneal astigmatism, suggesting that refractive correction can mitigate its visual impact.

Although several variables demonstrated trends toward association with CDVA, no independent predictors were identified on multivariable analysis. This likely reflects the relatively small sample size, which limit statistical power and increase variability. Similar challenges have been described in other real-world studies of secondary IOL fixation techniques [21].

The overall complication rate in this study was 10.3%, which is comparable to previously reported outcomes of the Yamane technique. Postoperative complications such as ocular hypertension, iris capture, and cystoid macular edema are well documented in the literature [6, 20]. In our cohort, 5.1% of patients developed ocular hypertension, which is similar to the 5.7% reported by Yamane et al. Iris capture has been reported to occur in approximately 8%–14.8% of cases. In contrast, our study demonstrated a lower incidence of 2.6% [6, 20, 22]. Importantly, no cases of severe complications, including endophthalmitis, retinal detachment, or haptic exposure, were observed during the follow-up period, supporting the short-term safety profile of this procedure.

Currently, three distinct approaches are utilized to guide needle tunneling during the Yamane SFIOL procedure. The first is the conventional freehand measurement using Castroviejo calipers, which is widely accessible but highly dependent on surgeon experience and susceptible to angular variability. The second is Yamane’s original double-needle stabilizer, a sophisticated device that standardizes both the distance from the limbus and the needle insertion angles (20° from the corneal limbus and 10° from the iris plane). Finally, the proposed Miri 4-Points Marker serves as an accessible alternative. It provides the flexibility to accommodate different corneal diameters and allows for the use of an anterior chamber maintainer, while being highly cost-effective for public healthcare settings. However, unlike the double-needle stabilizer, the Miri 4-Points Marker does not mechanically guide the angulation of the needle tunnel and still requires manual conjunctival marking by the surgeon.

This study contributes to the existing literature by providing the regional data on flanged intrascleral IOL fixation in Malaysia and by evaluating a novel marking device designed to improve surgical precision. The inclusion of multiple surgeons and real-world clinical scenarios enhances the generalizability of the findings. Furthermore, this study addresses a gap in the literature regarding procedural refinements of the Yamane technique, particularly in relation to marking strategies, as highlighted in recent reviews [23].

The Miri 4-Points Marker offers several practical advantages. By simplifying the marking process and reducing reliance on sequential measurements, it may reduce technical variability. These benefits are particularly relevant in high-volume or resource-limited settings, where access to advanced intraoperative guidance systems may be limited. Additionally, the device may serve as a useful adjunct in surgical training to facilitate consistent sclerotomy placement. Ophthalmologists’ experience with the Miri 4-Points Marker were also assessed using ‘System usability Scale’ (SUS), an end-user centred, standardized, 10-item Likert scale questionnaire covering ease of use and user confidence. The SUS recorded self-reported responses on a range from 1 (strongly disagree) to 5 (strongly agree) [24, 25]. Scoring was performed according to standard guidelines: for odd-numbered items (namely questions 1, 3, 5, 7 and 9), 1 was subtracted from the raw score. On the other hand, for even-numbered items (namely questions 2, 4, 6, 8 and 10), the raw score was subtracted from 5. The adjusted item scores were summed to yield a total score ranging from 0 to 40, which was then multiplied by 2.5 to obtain a final SUS score ranging from 0 to 100, with higher scores indicating better usability. All four ophthalmologists had good feedback with excellent mean SUS score of 89.4 [comprising of 75.0 (grade B), 85.0 (grade A+), 97.5 (grade A+) and 100 (grade A+)] as shown in Appendix.

This study’s strengths include the use of standardized surgical techniques that reflect real-world clinical practice. Rigorous statistical analyses, including univariable and multivariable regression with continuity correction for zero cell counts, further enhance the reliability of the findings.

Nevertheless, the retrospective design, relatively short-term follow-up, and performance of surgeries by four different ophthalmologists may introduce potential bias. This should be considered alongside a recent Malaysian study reporting visual outcomes at 6 weeks postoperatively (described as ultra-short follow-up), which further supports the reporting of short-term outcomes in this context [26]. On top of that, all surgeons were presumed to have gained more experience in performing Yamane SFIOL following the introduction of the Miri 4-Points Marker compared to the earlier period when Castroviejo calipers were used during the initial learning curve. However, no time-based data on the surgeons’ learning curve was captured or analysed, and this may represent a potential confounding factor that should be taken into consideration. As surgeons may have accumulated greater experience with the Yamane SFIOL technique over time, any benefit attributable to increased surgical proficiency would be expected to favour the Miri 4-Points Marker group. Therefore, the comparable clinical outcomes observed between groups should not be interpreted as evidence of equivalence between the two marking methods. As surgeon-specific learning curve and case-sequence data were not available, the influence of temporal improvements in surgical experience could not be quantified.

The relatively small sample size may limit the statistical power to detect subtle differences between the Miri 4-Points Marker group and the Castroviejo calipers group. Although larger cohorts are ideal for multivariable analyses, small retrospective studies of intrascleral IOL fixation in a single hospital setting have successfully evaluated visual outcomes and surgical factors with limited sample sizes. For instance, long-term good visual outcomes were reported in 12 eyes receiving IOL fixation with modified Yamane technique [27], demonstrating the potential feasibility of analyzing predictors such as corneal astigmatism or ocular comorbidities in exploratory regression models. Based on these precedents, our study included 32 eyes that underwent intrascleral lens fixation using the Miri 4-Points Marker, providing sufficient data to assess univariable associations with postoperative UCVA while acknowledging the limited power for detecting small effect sizes in multivariable analyses. Another limitation of this study is that direct measurements of conjunctival marking accuracy and reproducibility, such as inter-point distances, angular deviations, or objective assessments of IOL tilt and decentration, were not performed. Therefore, while the present findings support the short-term safety and feasibility of the Miri 4-Points Marker, its proposed advantage in improving marking precision remains to be directly evaluated in future studies.

## Conclusions

The Miri 4-Points Marker demonstrates comparable clinical outcomes to conventional Castroviejo calipers in terms of visual outcomes, postoperative complications and duration of operation. High corneal astigmatism was associated with poorer UCVA, while no independent predictors of CDVA were identified on multivariable analysis. These findings suggest that the Miri 4-Points Marker is a feasible and safe adjunct for conjunctival marking in Yamane intrascleral IOL fixation. Observed short-term outcomes were similar to caliper-based marking, although the chronological design precludes any conclusion or equivalence. Further studies with longer follow-up are warranted to evaluate long-term visual outcomes, IOL stability, and complication rates.

## Data Availability

All data and materials gathered during this study are included in this study.

## Appendix

‘System usability Scale’ (SUS) for ophthalmologists using the Miri 4-Points Marker:

**Table:**

Ophthalmologist	Q1	Q2	Q3	Q4	Q5	Q6	Q7	Q8	Q9	Q10	SUS Score
1	4	2	4	2	4	2	4	2	4	2	75.0
2	5	1	5	1	5	1	5	1	5	1	100
3	5	1	5	1	5	1	5	1	4	1	97.5
4	5	3	5	3	4	2	5	1	5	1	85.0
**Mean**											**89.4**

Q = Question

## Abbreviations

IOL: Intraocular lens
ACIOL: Anterior chamber intraocular lens
IFIOL: Iris-fixated intraocular lens
SFIOL: Scleral fixated intraocular lens
ICCE: Intracapsular cataract extraction
ECCE: Extracapsular cataract extraction
UCVA: Uncorrected visual acuity
CDVA: Corrected distant visual acuity
SUS: System usability scale
ELP: Effective lens position
